# Baicalin Weakens the Porcine ExPEC-Induced Inflammatory Response in 3D4/21 Cells by Inhibiting the Expression of NF-*κ*B/MAPK Signaling Pathways and Reducing NLRP3 Inflammasome Activation

**DOI:** 10.3390/microorganisms11082126

**Published:** 2023-08-21

**Authors:** Bingbing Zong, Yong Xiao, Mingxing Ren, Peiyi Wang, Shulin Fu, Yinsheng Qiu

**Affiliations:** 1 Hubei Key Laboratory of Animal Nutrition and Feed Science, Wuhan Polytechnic University, Wuhan 430023, China; 2 Engineering Research Center of Feed Protein Resources on Agricultural By-Products, Ministry of Education, Wuhan Polytechnic University, Wuhan 400023, China; 3 Hubei Collaborative Innovation Center for Animal Nutrition and Feed Safety, Wuhan 400023, China

**Keywords:** porcine ExPEC, baicalin, 3D4/21, cell inflammatory responses

## Abstract

Porcine extraintestinal pathogenic *Escherichia coli* (ExPEC) is a leading cause of death in pigs and has led to considerable economic losses for the pig industry. Porcine ExPEC infections often cause systemic inflammatory responses in pigs, characterized by meningitis, arthritis, pneumonia, and septicemia. Baicalin has been reported to possess potent anti-inflammatory activity, but its function in porcine ExPEC remains unknown. The aim of this study was to explore the protective effect and mechanism of baicalin against the porcine ExPEC-induced inflammatory responses in 3D4/21 cells. After treatment with baicalin, the effects on cell damage, the level of pro-inflammatory cytokines, the expression of nuclear factor-κB (NF-κB)/mitogen-activated protein kinase (MAPK) signaling pathways, and the activation of NOD-like receptor protein 3 (NLRP3) inflammasomes were examined. Our results show that baicalin significantly reduced the damage to 3D4/21 cells infected with porcine ExPEC PCN033. Further study showed that baicalin significantly reduced the transcription and expression of pro-inflammatory cytokines such as interleukin-1β (IL-1β), interleukin-6 (IL-6), and interleukin-8 (IL-8). Furthermore, baicalin inhibited the phosphorylation of proteins such as P65, nuclear factor κB inhibitor α (IκBα), extracellular regulated kinase (ERK), c-Jun N-terminal kinase (JNK), and P38 and reduced the expression levels of proteins such as NLRP3, apoptosis-associated speck-like protein containing a CARD (ASC), and caspase-1. These results reveal that baicalin reduced the damage to 3D4/21 cells by inhibiting the expression of NF-κB/MAPK signaling pathways and blocking NLRP3 inflammasome activation in 3D4/21 cells infected with porcine ExPEC. Taken together, these results suggest that baicalin may have potential as a medicine for the treatment of porcine ExPEC-infected pigs by regulating inflammatory responses. This study provides a novel potential pharmaco-therapeutic approach to preventing porcine ExPEC infection.

## 1. Introduction

Extra-intestinal pathogenic *Escherichia coli* (ExPEC) is one of the groups of *Escherichia coli* which has specific virulence factors [1]. Usually, ExPEC is harmless to the intestine. However, once ExPEC is transferred to other organs from the intestine, it can infect the tissues of the distal intestinal tract, causing life-threatening diseases [2,3]. ExPEC includes neonatal meningitis-causing *E. coli* (NMEC), uropathogenic *E. coli* (UPEC), avian pathogenic *E. coli* (APEC), and septicemic *E. coli* (SEPEC) [2,4,5]. In China in 2011, there was an outbreak of acute sepsis, pneumonia, and meningitis caused by porcine ExPEC in large-scale pig farms [6]. This outbreak could be considered the first outbreak of porcine ExPEC in China. Porcine ExPEC, being a relatively novel pathotype of ExPEC, shares a common feature with other ExPECs from humans or avian species. This common feature is the ability to cause bloodstream infections, which ultimately leads to septicemia [7]. Moreover, some studies have reported similar virulence profiles and serogroups between porcine and human ExPECs, suggesting that porcine ExPEC has zoonotic potential [6,8,9,10]. ExPEC is multidrug-resistant and can cause various diseases in humans and animals [11,12,13], such as urinary tract infections, newborn meningitis, peritonitis, bacteremia, and septicemia [2,3,4,14,15]. Notably, ExPEC infections occur worldwide and lead to great economic losses [2].

Porcine ExPEC is an important pathogen threatening the porcine industry and has caused major economic losses for the global pig industry [16]. ExPEC infection can cause meningitis, arthritis, pneumonia, and septicemia in pigs [6,17,18]. With the rapid development of the porcine industry in China, the isolation rate of porcine ExPEC has been increasing year on year. In addition, the drug resistance of ExPEC has increased substantially, especially with the emergence of multidrug-resistant *E. coli*, which has posed a serious challenge to the prevention and control of porcine ExPEC infections [6,18]. However, there are currently no effective prevention methods or treatments for porcine ExPEC infections. Therefore, it is necessary to study the pharmaco-therapeutic approach of porcine ExPEC for effective prevention and control of the diseases caused by porcine ExPEC.

Antibiotics are widely applied in clinics for the treatment of infections caused by ExPEC. However, the misuse of antibiotics frequently leads to antibiotic resistance. Many reports have shown that the prevalence of multi-antibiotic *E. coli* has increased markedly in animals [17,19,20]. Therefore, new prophylactic and therapeutic approaches are urgently needed to treat bacterial diseases by overcoming bacterial resistance. In recent years, naturally occurring compounds isolated and developed from medicinal plants have attracted a resurgence in interest [21]. Furthermore, numerous Chinese herbal medicines have been widely used in clinical cases and are useful in the treatment of inflammatory diseases, including mastitis, acute lung injury, and endometritis [22].

**Figure 1:**
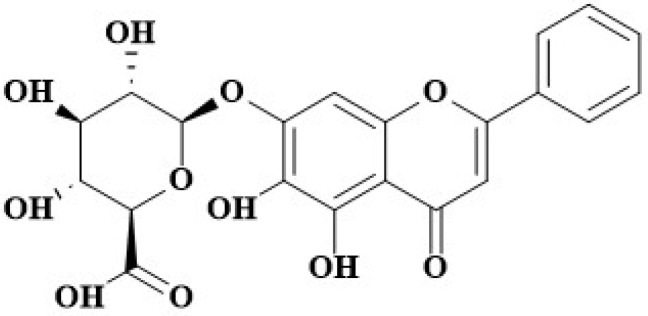
The structure of BA.

Baicalin (BA; Figure 1), namely baicalein 7-O-β-D-glucuronide, is a flavonoid compound isolated from medicinal plants such as *Scutellaria baicalensis* [23]. Multiple studies have highlighted the robust biological activities and significant functions of BA, such as its antimicrobial and antioxidant activities [24]. For instance, it has been demonstrated to possess anti-inflammatory, antioxidant, and anticancer effects on organs like the lungs and the uterus [25,26]. BA can inhibit biofilm formation, attenuate quorum-sensing controlled virulence, and enhance *Pseudomonas aeruginosa* clearance in mice [27]. BA has been proven to possess inhibitory effects on virulence phenotypes in *Pseudomonas aeruginosa* and APEC [28,29].

In recent years, an increasing number of works in the literature have reported the effects of BA on inflammatory diseases. For example, BA reduced mastitis caused by *Staphylococcus aureus*, inhibited cerebral ischemia, and prevented encephalitis [30,31,32]. BA reduced A549 cell injury induced by *Staphylococcus aureus* and protected mice from *S. aureus* pneumonia [33,34]. BA improved the survival of mice with polymicrobial sepsis by suppressing the inflammatory response and lymphocyte apoptosis [35]. It has been applied in the treatment of various inflammatory diseases, including periodontitis, hepatitis, and ulcerative colitis [36,37,38]. However, the effects and protective mechanisms of BA regarding porcine ExPEC are still unclear.

Based on previous reports, we speculate that BA may be utilized as a potential therapeutic agent to control porcine ExPEC infections. In this study, we investigate the effects of BA on the damage to and inflammatory responses in 3D4/21 cells infected with porcine ExPEC and attempted to investigate the effects of BA on the pathogenesis of porcine ExPEC and its underlying molecular mechanisms.

## 2. Materials and Methods

### 2.1. Reagents

BA (≥98%) was purchased from Sichuan Xieli Pharmaceutical Co., Ltd. (Chengdu, China, C031B180101). Luria–Bertani (LB) medium was prepared with yeast extract (OXOID, LP0021), tryptone (OXOID, LP0042B), and sodium chloride (Shanghai Test, 10019318). Fetal bovine serum (Gbico, 1456384) and trypsin (Gibco, 2520-056) were obtained from Gibco (Invitrogen S.r.l., Milan, Italy). DMEM with high glucose was obtained from Gibco (Hyclone, SH30243.01). RNA and qPCR reagents were obtained from Vazyme (Nanjing, China). Finally, ELISA kits were purchased from 4A Biotech (Beijing, China). The NLRP3 antibody was purchased from proteintech (Wuhan, China), and other antibodies were obtained from CST (Boston, MA, USA). All other chemicals were of reagent grade.

### 2.2. Strains and Growth Conditions

The porcine ExPEC PCN033 strain was donated to us by the Key Lab of Preventive Veterinary Medicine in Hubei Province. *E. coli* strains were grown in Luria–Bertani (LB) broth or on LB agar plates containing antibiotics of specific concentrations. The primers used in this study are listed in Table 1.

### 2.3. Culture of Porcine Alveolar Macrophage 3D4/21

Porcine alveolar macrophage (3D4/21) cells were preserved in our laboratory. Cell lines (3D4/21) were grown in Dulbecco’s modified eagle medium with high glucose (DMEM; Hyclone) supplemented with 10% heat-inactivated fetal bovine serum (FBS; Gbico) and 1% MEM non-essential amino acids (Gibco). The cell lines were grown at 37 °C in a humidified atmosphere of 5% CO${}_{2}$.

### 2.4. Determination of the Minimum Inhibitory Concentration (MIC) of BA

The MIC of BA to PCN033 was determined by the standard CLSI broth microdilution method. BA was dissolved in LB broth and diluted to 50, 100, 200, 400, 800, 1600, and 3200 μg/mL. The PCN033 was diluted to 1 × $10^{6}$ CFU/mL with LB broth. Bacterial liquid and BA were added to 96-well plates at a ratio of 1:1. (The final concentrations were 25, 50, 100, 200, 400, 800, and 1600 μg/mL.) The turbidity was observed after incubation at 37 °C for 4 h.

### 2.5. Growth Characteristic Analysis

In order to investigate the effect of BA on the growth characteristics of PCN033, the growth characteristics of PCN033 in LB liquid medium and LB liquid medium with different concentrations of BA were measured within 24 h. In brief, a single colony of PCN033 was cultured overnight in 10 mL of LB liquid medium (37 °C, 180 rpm). On the second day, overnight bacterial cultures were transferred to normal, fresh LB liquid medium or fresh LB liquid medium containing different concentrations of BA at a ratio of 1:1000. Samples were grown at 37 °C, accompanied by shaking at 180 rpm for 12 h. $OD_{600}$ was determined, and the colony-forming units (CFUs) were counted every hour as described previously [39].

### 2.6. Cell Viability Assay with CCK8

The effect of BA on cell viability was evaluated by using a CCK-8 kit (Dojindo, Beijing, China). The confluent monolayers of the 3D4/21 cells were used to determine the viability of the cell. The 3D4/21 cells were seeded in a 96-well plate (1 × $10^{5}$ cells/mL, 100 μL) and cultured overnight. On the second day, the supernatant was discarded, and the cells were washed once with the preheated PBS. A total of 200 μL BA solution (25 μg/mL, 50 μg/mL, 100 μg/mL, and 200 μg/mL) was added to the preheated PBS. At the same time, control wells (containing only cells) and blank wells (containing only culture medium) were set. After incubation for 3 h, 10 μL CCK-8 solutions were added to all wells. After co-incubation for 1.5 h, the absorbance of the supernatant at 450 nm was measured using a microplate reader, where cytotoxicity = (test well absorbance − blank well absorbance)/(control well absorbance − blank well absorbance).

### 2.7. Lactate Dehydrogenase (LDH) Activity

The cytotoxicity of PCN033 to 3D4/21 cells was tested by lactate dehydrogenase assays in 96-well plates as described previously, with a few modifications [40]. Cells were seeded into 96-well plates with 1 × 10${}^{4}$ cells in each well, and monolayer cells were obtained via overnight culture. The overnight bacterial cultures were transferred to fresh LB liquid medium containing different concentrations of BA (25, 50, and 100 μg/mL) at a ratio of 1:1000. After co-culturing for 4 h, the monolayer cells were washed three times with the preheated PBS to remove the residual antibiotics. Bacteria in the stationary phase were added to the 96-well plates at an infection ratio of 10:1 (bacteria/cells) for 90 min. The LDH activity was then determined by using Beyotime LDH-cytotoxicity colorimetric assay kits (catalog number C0016). The LDH activities were expressed according to the following formula: Cell mortality (%) = (Test Sample − Low Control) / (High Control − Low Control) × 100%.

### 2.8. Expression of Inflammatory Genes by q-PCR

The test was divided into three groups, including the control group, the PCN033 group, and the PCN033 + BA treatment group (25, 50, and 100 μg/mL). The cells were pretreated with BA for 1 h and then co-cultured with the number of bacteria with an MOI of 10. The RNA was extracted with Total RNA Extraction Reagent (Vazyme, Nanjing, China, R401-01-AA), and then the RNA was reverse transcribed to cDNA using a Hiscript II® 1st Strand cDNA Synthesis Kit (Vazyme, R212-02) with oligo (dT) primers (Tsingke Biotechnology Co., Ltd., Wuhan, China). The transcriptional levels of the inflammatory genes were tested by using qRT-PCR (Taq Pro Universal SYBR qPCR Master MIX, Vazyme, Q712-02). The gene β-actin served as the housekeeping gene and the untreated PCN033 and cells as reference samples. The primers for real-time PCR are listed in Table 1.

### 2.9. Measurement of the Cytokines

To determine the roles of BA in the PCN033-induced inflammation response in 3D4/21 cells, the secretion levels of pro-inflammatory factors were determined by ELISA. The cells were pretreated with BA (25, 50, or 100 μg/mL) for 1 h and then co-cultured with a number of PCN033 with an MOI of 10 for 3 h. The culture supernatants were then collected. The pro-inflammatory cytokines IL-6, IL-8, and IL-1β were measured by ELISA kits (Beijing 4A Biotechnology Co., Ltd., Beijing, China) following the manufacturer’s instructions. Finally, the $OD_{450}$ absorbance of each sample was determined.

### 2.10. Western Blot Assay

Western blotting was used to measure the activation level of inflammatory signal pathways. The cell samples were dissociated by an RIPA lysis buffer (Beyotime Biotechnology Co., Ltd., Nantong, China, P0013) and centrifuged at 12,000× *g* for 10 min at 4 °C. Then, the cell lysates were fractionated on 12% SDS-PAGE and transferred to PVDF membranes. These PVDF membranes were blocked with 5% BSA for 3 h and then incubated with a special primary antibody (1:500 dilution) at 4 °C overnight. Afterward, the membranes were incubated with the corresponding HRP-labeled secondary antibodies (1:25,000 dilution) at room temperature for 3 h and then washed three times with a TBST buffer. The immunoreactive proteins were detected by using an enhanced chemiluminescence Western blotting detection kit. The protein GAPDH or β-actin served as an internal control.

### 2.11. Statistical Analysis

All assays were performed in triplicate at three different times, and the data were analyzed using GraphPad Prism 6 (2) (San Diego, CA, USA). Multiple comparisons were analyzed through ANOVA. A *p* value of < 0.05 was considered significantly different.

## 3. Results

### 3.1. The Antibacterial Effect of BA on PCN033

**Figure 2:**
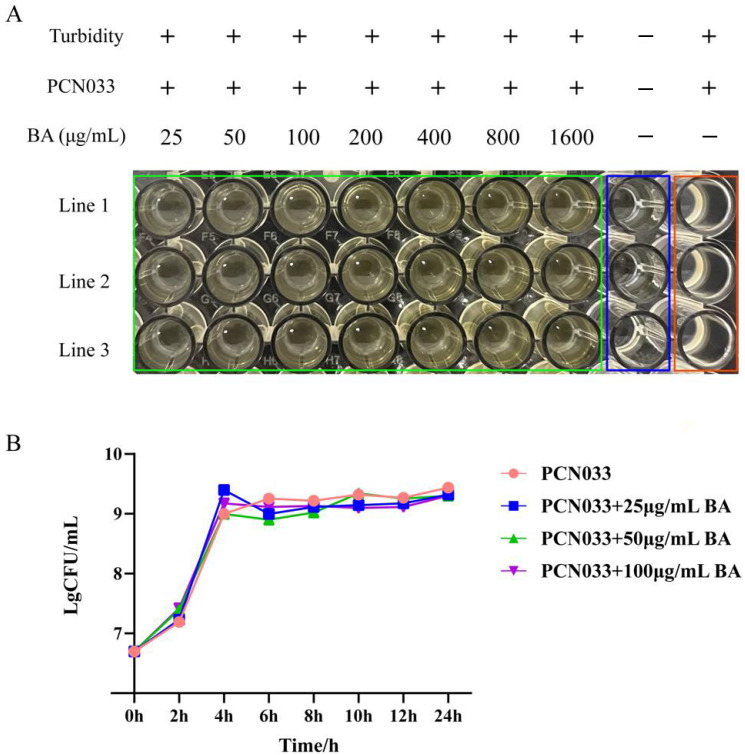
The antibacterial effect of BA on PCN033. (**A**) The MIC of BA on PCN033. (**B**) The growth curve of PCN033 with different concentrations of BA.

In order to explore the antibacterial effect of BA on porcine PCN033, the MIC of BA and the effect of BA on the growth curve of PCN033 were determined. As shown in Figure 2A, BA (25–1600 μg/mL) had no inhibitory effect on the growth of PCN033. Thus, the MIC of BA against PCN033 was over 1600 μg/mL. Further research results (Figure 2B) showed that BA (25–100 μg/mL) had no effect on the growth curve of PCN033. These results suggest that BA does not affect the growth of PCN033 within a concentration of 100 μg/mL (Figure 2A,B).

### 3.2. Effects of BA on the Damage to 3D4/21 Cells Infected with PCN033

**Figure 3:**
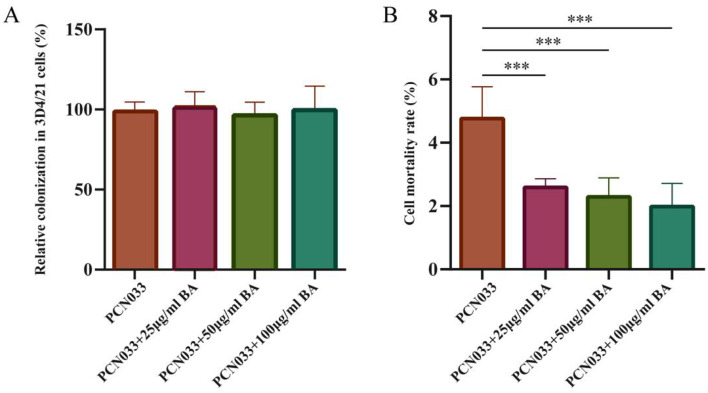
The effect of BA on cell damage caused by PCN033. (**A**) The relative colonization rates of PCN033 in 3D4/21 cells. Rates of colonized numbers are expressed relative to those of PCN033 (100%). These experiment results show that the colonized number of PCN033 treated with BA was significantly lower than that for PCN033. (**B**) The cytotoxicity activities of PCN033 and PCN033 treated with BA with 3D4/21 cells. Rates of cytotoxicity activities are expressed relative to those of PCN033 (100 %). Data are presented as means ± SD of three independent experiments performed in triplicate. These experiment results show that the cytotoxicity activities of PCN033 treated with BA for 3D4/21 cells were significantly lower than those of PCN033 with 3D4/21 cells (*** *p* < 0.001).

To investigate the effect of BA on the cell damage induced by PCN033, the cytotoxicity abilities of PCN033 on 3D4/21 cells with or without (set as 100%) BA treatment were compared in vitro. Before comparing the cytotoxicity abilities, the time point at which BA does not affect cell proliferation was determined. After co-incubation with 3D4/21 cells for 90 min, the results showed that no significant number of PCN033 cells treated with BA was colonized compared with PCN033 (Figure 3A). The cell mortality rate results showed that the cytotoxicity activities of the BA-treated group were significantly lower than those of the PCN033-infected group (Figure 3B, *p* < 0.001).

### 3.3. Effects of BA on the Viability of 3D4/21 Cells

**Figure 4:**
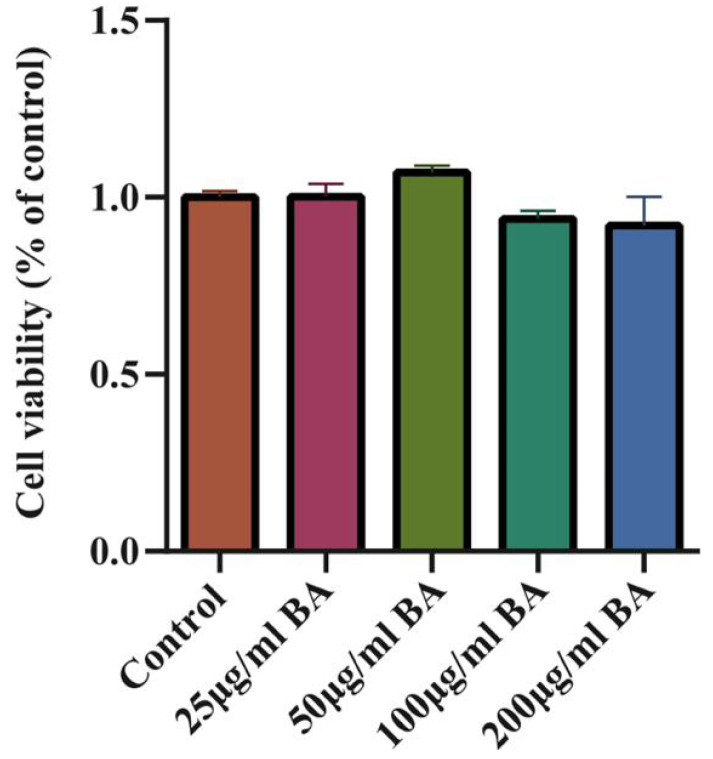
The effect of BA on 3D4/21 cell viability and the toxic effect of BA on 3D4/21 cells (3 h). The results show that BA (25–200 μg/mL) had no effect on the cell viability of 3D4/21 cells.

Whether BA had a toxic effect on cells was analyzed by a CCK-8 assay. The results showed that BA (25–200 μg/mL) had no effect on the cell viabilities of 3D4/21 cells (Figure 4).

### 3.4. Effects of BA on the Transcription Levels of Inflammatory Factors in 3D4/21 Cells Infected with PCN033

**Figure 5:**
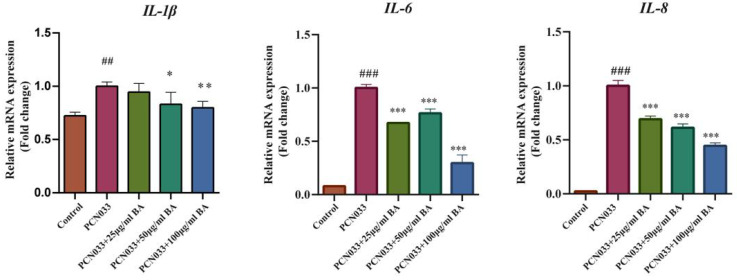
The effects of BA on the transcription levels of PCN033-induced inflammatory factor production in 3D4/21 cells and the transcription levels of inflammatory factors IL-1β, IL-6, and IL-8 (3 h). The results show that the transcription level of inflammatory factors IL-1β, IL-6, and IL-8 were increased significantly after PCN033 infection and decreased significantly after treatment with BA (## *p* < 0.01 vs. control; ### *p* < 0.001 vs. control; * *p* < 0.05; ** *p* < 0.01; *** *p* < 0.001).

Whether BA had an effect on the inflammatory factors was analyzed with qRT-PCR. The qRT-PCR results show that the transcription levels of the inflammatory factors IL-1β, IL-6, and IL-8 were significantly increased after PCN033 infection, while the transcription levels of the inflammatory factors IL-1β, IL-6, and IL-8 were significantly decreased after treatment with BA (Figure 5).

### 3.5. Effects of BA on the Expression Levels of Inflammatory Factors in 3D4/21 Cells Infected with PCN033

**Figure 6:**
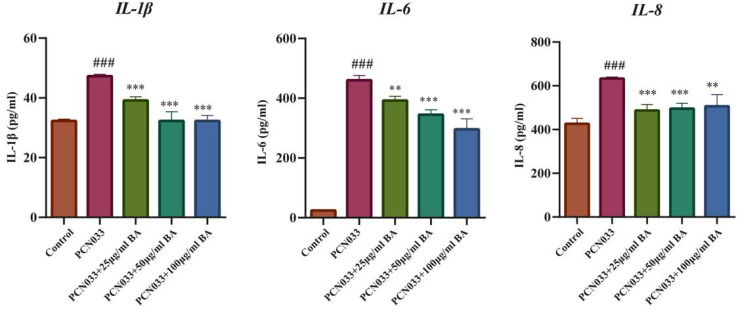
The effects of BA on the expression levels of inflammatory factors induced by PCN033 and the expression levels of inflammatory factors IL-1β, IL-6, and IL-8 (3 h). The ELISA results show that the expression levels of the inflammatory factors IL-1β, IL-6, and IL-8 increased significantly after PCN033 infection and decreased significantly after treatment with BA (### *p* < 0.001 vs. control; ** *p* < 0.01; *** *p* < 0.001).

Whether BA had an effect on the inflammatory factors was further analyzed through ELISA. The ELISA results demonstrate that the expression levels of the inflammatory factors IL-1β, IL-6, and IL-8 increased significantly in the 3D4/21 cells infected with PCN033 and decreased significantly in PCN033-infected cells after treatment with BA (Figure 6), which was consistent with the qRT-PCR experiment results.

### 3.6. Effect of BA on the NF-κB Signaling Pathway in 3D4/21 Cells Infected with PCN033

**Figure 7:**
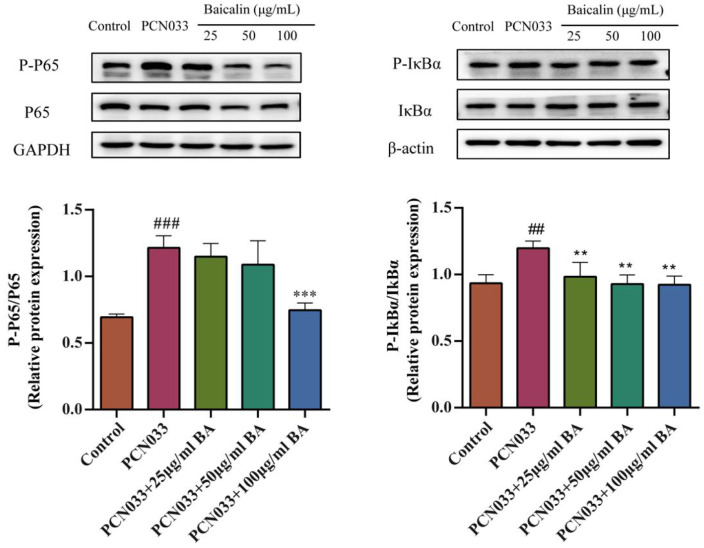
The effects of BA on the PCN033-induced NF-κB signaling pathway and the protein expression levels of the NF-κB signaling pathway (8 h). The results show that the levels of phosphorylated p65 and IκBα were significantly elevated in the PCN033 group compared with those of the control group. However, BA significantly inhibited the levels of phosphorylated p65 and IκBα induced by PCN033 (## *p* < 0.01 vs. control; ### *p* < 0.001 vs. control; ** *p* < 0.01; *** *p* < 0.001).

The NF-κB signaling pathway plays an important role in inflammatory response. In order to investigate whether BA had an effect on the inflammatory response in PCN033-infected cells, the protein expression levels of the NF-κB signaling pathway were tested by Western blotting. These results show that the levels of phosphorylated NF-κB p65 and IκBα in the PCN033 group were significantly increased in comparison with those of the control group. However, BA significantly inhibited the levels of phosphorylated NF-κB p65 and IκBα in the 3D4/21 cells infected with PCN033 (Figure 7).

### 3.7. Effect of BA on the MAPK Signaling Pathway in 3D4/21 Cells Infected with PCN033

**Figure 8:**
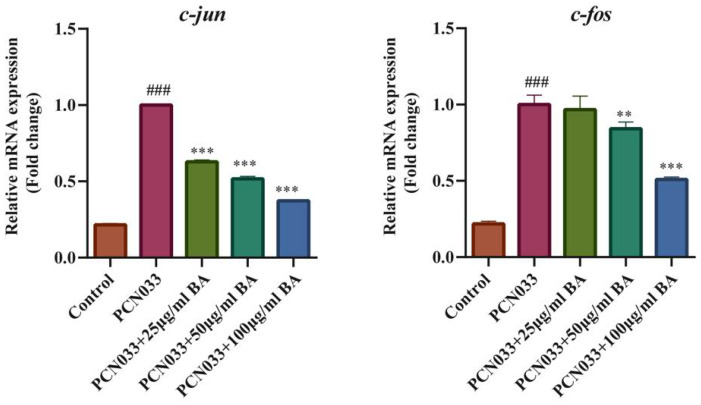
The transcription levels of the inflammatory factors c-jun and c-fos (3 h). The qRT-PCR results show that the transcription levels of the inflammatory factors c-jun and c-fos increased significantly after being infected with PCN033 and decreased significantly after treatment with BA (### *p* < 0.001 vs. control; ** *p* < 0.01; *** *p* < 0.001).

**Figure 9:**
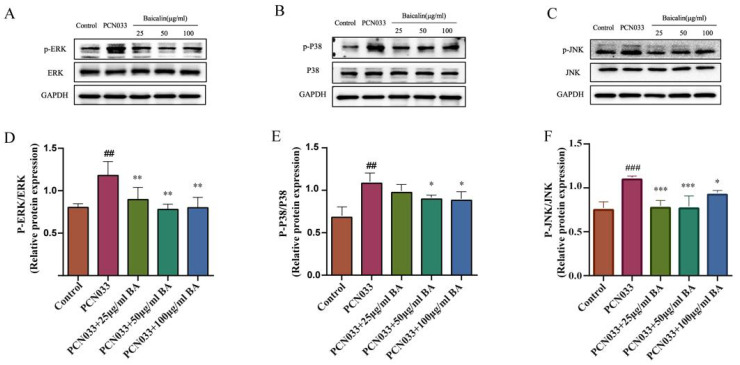
The effects of BA on PCN033-induced MAPK signaling pathways. (**A**,**D**) The levels of phosphorylated ERK (8 h). (**B**,**E**) The levels of phosphorylated P38 (8 h). (**C**,**F**) The levels of phosphorylated JNK (8 h). Western blot results show that the levels of phosphorylated ERK, P38, and JNK increased significantly after being infected with PCN033 and were inhibited significantly after treatment with BA (## *p* < 0.01 vs. control; ### *p* < 0.001 vs. control; * *p* < 0.05; ** *p* < 0.01; *** *p* < 0.001).

In order to investigate whether BA had an effect on the inflammatory response in PCN033-infected 3D4/21 cells, the protein expression level of the MAPK signaling pathway was further tested with qRT-PCR and Western blotting. The qRT-PCR results show that the transcription levels of the inflammatory factors c-jun and c-fos increased significantly after PCN033 infection and decreased significantly after treatment with BA (Figure 8). The Western blot results show that the expression levels of phosphorylated ERK, P38, and JNK in the PCN033 group significantly increased in comparison with those of the control group. However, BA significantly inhibited the levels of phosphorylated ERK, P38, and JNK in the 3D4/21 cells infected with PCN033 (Figure 9).

### 3.8. Effect of BA on the NLRP3 Inflammasome in 3D4/21 Cells Infected with PCN033

**Figure 10:**
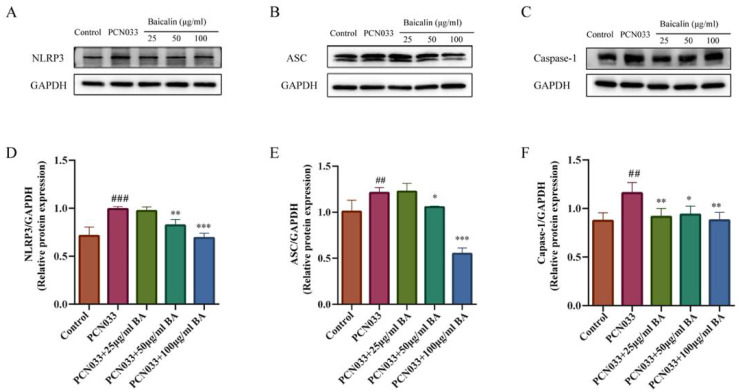
The effect of BA on the PCN033-induced NLRP3 inflammasome. (**A**,**D**) The expression of NLRP3 (8 h). (**B**,**E**) The expression levels of ASC (8 h). (**C**,**F**) The expression levels of phosphorylated Caspase-1 (8 h). The results show that the expressions of NLRP3, ASC, and Caspase-1 increased significantly after infection with PCN033 and were inhibited significantly after treatment with BA (## *p* < 0.01 vs. control; ### *p* < 0.001 vs. control; * *p* < 0.05; ** *p* < 0.01; *** *p* < 0.001).

The NLRP3 inflammasome plays an important role in inflammatory response. In order to investigate whether BA had an effect on the inflammatory response in PCN033-infected 3D4/21 cells, the protein expression levels of the NLRP3 inflammasome were further tested through Western blotting. The results show that the expression levels of NLRP3, ASC, and Caspase-1 in the PCN033 group were significantly increased in comparison with those of the control group. However, BA significantly inhibited the expression of NLRP3, ASC, and Caspase-1 in the PCN033-infected 3D4/21 cells (Figure 10).

## 4. Discussion

Porcine ExPEC has caused major economic losses in the porcine industry [16]. Porcine ExPEC can cause serious diseases in pigs, such as meningitis, pneumonia, arthritis, and septicemia [6,17,18]. The isolation rate of porcine ExPEC in Chinese farms has increased gradually, and 81.9–100% of isolated ExPEC exhibited multidrug resistance [6,17]. Therefore, new therapeutic approaches urgently need to be developed and used to treat diseases caused by porcine ExPEC.

BA (Figure 1), a flavonoid compound isolated from *Scutellaria baicalensis*, has important anti-inflammatory, anti-microbial, and anti-oxidant activities [23,24]. Recent studies have shown that BA has important anti-inflammatory, antioxidant, and anticancer roles [25,26]. Porcine ExPEC PCN033 has been reported to cause inflammatory lesions in multiple tissues and organs in mouse and pig models [6,41,42]. However, whether BA can inhibit porcine ExPEC PCN033-induced inflammatory responses has not been reported.

In order to study whether BA has an antibacterial effect on porcine ExPEC PCN033, we first tested the antibacterial effect of BA on PCN033. The results show that the MIC of BA against PCN033 was over 1600 μg/mL (Figure 2A). Furthermore, the growth characteristics of PCN033 in the control group were not significantly different in comparison with those of the BA (25–100 μg/mL) group (Figure 2B). It was demonstrated that BA had no effect on the growth characteristics of PCN033 within a concentration of 100 μg/mL, which is consistent with previous research results [29]. PCN033 infection can cause highly inflammatory lesions in both mouse and pig models [6,41]. BA has been reported to be effective against sepsis by alleviating the *Staphylococcus aureus*-induced inflammatory response in mice [23,43]. Therefore, we speculated that BA may attenuate PCN033 infection by inhibiting the inflammatory response. To test our hypothesis, we further studied the effect of BA on the damage to 3D4/21 cells infected with PCN033 and the anti-inflammatory effect of BA on 3D4/21 cells infected with PCN033.

The cell damage test showed that BA significantly inhibited damage to 3D4/21 cells caused by PCN033 infection (Figure 3A,B), which is consistent with previous reports [29]. An inflammatory response is the major pathological feature of ExPEC invasion [29]. Macrophages play an important role in regulation of the inflammatory response [44]. Activation of inflammation-associated signaling pathways can induce the expression of inflammatory cytokines such as IL-1β, IL-6, and IL-8 [45]. The qRT-PCR results demonstrate that BA significantly inhibited the transcription levels of inflammatory factors IL-1β, IL-6, and IL-8 in the 3D4/21 cells infected with PCN033 (Figure 5). BA’s inhibition of inflammatory factors was further confirmed by ELISA assays (Figure 6), which is consistent with previous research results [29].

Nuclear factor kappa-B (NF-κB) is a widely expressed nuclear transcription factor and is regarded as one of the most important regulators of the inflammatory process [46]. Activation of the NF-κB signaling pathway can promote the secretion of many inflammatory mediators and aggravate the inflammatory response [47]. Peng et al. [29] showed that BA can inhibit activation of the NF-κB signaling pathway when induced by APEC. Cui et al. [48] reported that BA can block the toll-like receptor (TLR) 4/NF-κB pathway in mice. Our results show that BA significantly inhibited the level of phosphorylated NF-κB p65 and IκBα induced by PCN033 (Figure 7), which is consistent with previous research results [29,48].

It has been reported that JNK is a potential target for the treatment of inflammatory diseases [49]. BA treatment reduced the high phosphorylation levels of c-Jun N-terminal kinase (JNK), p65, p-38, and ERK1/2 triggered by atherosclerosis [50]. Recent research reported that azelastine inhibited inflammation induced by LPS by inhibiting the JNK/NF-κB pathway [51], and curcumin derivative C66 suppressed inflammation through inhibition of the JNK pathway [52]. Our results show that BA significantly inhibited the transcription levels of inflammatory factors c-jun and c-fos in the 3D4/21 cells induced by PCN033 (Figure 8). At the same time, BA significantly inhibited the levels of phosphorylated ERK, P38, and JNK in the 3D4/21 cells induced by PCN033 (Figure 9). Our results are consistent with these reports [50,51,52].

NF-κB and MAPK signaling are critical for activation of the NLRP3 inflammasome [53,54]. BA can inhibit inflammation by downregulating the NF-κB/MAPK signaling pathway and inhibiting activation of the NLRP3 inflammasome simultaneously [53,55]. Our studies show that the transcription and secretion level of IL-1β were inhibited. The maturation of IL-1β depends on the activation of pro-caspase-1, and the processing and secretion of IL-1β are closely related to NLRP3 in response to various pathogens. Our studies also demonstrated that BA inhibited the inflammation response by suppressing the NF-κB pathway and the JNK and MAPK signaling pathways. Activation of the NLRP3 inflammasome is involved in many inflammatory diseases [54]. The NLRP3 inflammasome is composed of NLRP3, ASC, and pro-caspase-1 [54]. In our further study, BA significantly inhibited the protein expression levels of NLRP3, ASC, and caspase-1 (Figure 10). These results indicate that BA is capable of inhibiting the activation of the NLRP3 inflammasome.

## 5. Conclusions

In conclusion, our findings demonstrate that BA may exert its anti-inflammatory activity by inhibiting the NF-κB pathway and suppressing activation of the JNK and NLRP3 signaling pathways. This study provides a new route for the prevention and treatment of porcine ExPEC.

## Figures and Tables

**Table 1:** List of primers used in this study.

Primer	Sequence	Remark
β-actin	TGCGGGACATCAAGGAGAAG	Forward
	AGTTGAAGGTGGTCTCGTGG	Reverse
IL-6	TGTCGAGGCTGTGCAGATTAGT	Forward
	CATCCATCGTTCTGTGACTGC	Reverse
IL-8	ACAGCAGTAACAACAACAAG	Forward
	GACCAGCACAGGAATGAG	Reverse
IL-1β	GCTGGAGGATATAGACCCC	Forward
	GTTGGGGTACAGGGCAGAC	Reverse
c-jun	AGAATCCGAAGGGAAAGGA	Forward
	CTTCTCCTTCAGCAGGTTGG	Reverse
c-fos	GCTGACAGATACACTCCAAGCGG	Forward
	AGGAAGACGTGTAAGTAGTGCAG	Reverse

## Data Availability

The datasets used or analyzed during the current study are available from the corresponding authors upon reasonable request.
